# Retraction: Drug-Eluting Stents for Acute Coronary Syndrome: A Meta-Analysis of Randomized Controlled Trials

**DOI:** 10.1371/journal.pone.0160297

**Published:** 2016-07-26

**Authors:** The PLOS ONE Editors

Lishan Wang requested the retraction of this article.

An institutional investigation conducted by the Administration Office of Bio-X Institutes, Shanghai Jiao Tong University, indicated that Lishan Wang was solely responsible for the article, contrary to information provided to the journal at time of submission.

In line with the author’s request and the recommendation by Shanghai Jiao Tong University, the *PLOS ONE* Editors retracted the research article [1] on July 26, 2016.

This retraction notice and the linked article [1] were republished on June 7, 2022 to amend content, references, and metadata.
